# DORA Editorial

**DOI:** 10.1099/mgen.0.000238

**Published:** 2018-12-05

**Authors:** Tanya Parish, Mark Harris, Norman Fry, Kalai Mathee, Martha E. Trujillo, Stephen Bentley, Nicholas Thomson

**Affiliations:** ^1^​ Infectious Disease Research Institute, Seattle, WA, USA; ^2^​ University of Leeds, Leeds, UK; ^3^​ Public Health England, London, UK; ^4^​ Florida International University, Miami, FL, USA; ^5^​ University of Salamanca, Salamanca, Spain; ^6^​ Wellcome Sanger Institute, Cambridge, UK

**Keywords:** DORA, Impact Factor, metrics, editorial

The Editors, Senior Editors and Editors-in-Chief of the Society journals (*Microbiology*, *Journal of General Virology*, *Journal of Medical Microbiology*, *International Journal of Systematic and Evolutionary Microbiology*, *Microbial Genomics* and *Access Microbiology*) are committed to serving the microbiology community by providing access to read and publish the broadest range of papers in our fields. We are continually reviewing the nature and scope of our journals and considering ways in which we can serve our authors, our readership and the society. One of the areas of concern to our community has been the improper use of Journal Impact Factors in assessing the quality or impact of science and scientists. There has been a long debate over appropriate ways to monitor the quality of science that is funded and published and while there is no consensus on the most effective or efficient methods, it is clear that judging the quality of individual pieces of work based on the impact factor of the journal in which they are published is not useful or appropriate.

As noted, Impact Factors arose as a means for libraries to prioritize journals subscriptions and not as a means of assessing the quality of individual works [The Declaration on Research Assessment (DORA), https://sfdora.org/]. A great deal has been written about the limited utility of the Impact Factor in evaluating the scientific merit or the impact of a piece of work in its field. Differences in the size of a community or area of study, the lack of measurement of long-term implications and biases in citations towards reviews over primary papers for expediency are some of the factors that we consider to be problematic. Besides, irreversible damage can be done to the careers of scientists by the improper use of these metrics.

The Microbiology Society journals have been using a variety of alternative metrics, mostly focused on individual papers (see altmetrics, for example doi: 10.1099/mic.0.000692) to provide a different and, we hope, more relevant means of assessing and accessing information on the manuscript level. As an extension of this, the Society has recently become a signatory to DORA, the San Francisco Declaration on Research Assessment. DORA challenges the role played by the Impact Factor and promotes the need to improve the ways in which the outputs of scholarly research are evaluated; it promotes the assessment of research on its own merits rather than on the basis of the journal in which the research is published.

## A message from the Editors-in-Chief

Jodi Lindsay, Chair of the Microbiology Society Publishing Committee. ‘The health and prosperity of our Microbiology Society community relies on the responsible use of publishing metrics. Each time we use metrics to hire, promote, referee, review or collaborate has an impact. Individual paper citation metrics have clear advantages over journal impact factors for assessing the quality of a publication.’

Tanya Parish. ‘We removed the Impact Factor from *Microbiology*’s home page some time ago, as we felt this was not a useful metric for our authors or readers. We welcome this (signing DORA) as another step towards eliminating misleading metrics and improving scientific discourse and dissemination.’

Kalai Mathee and Norman Fry. ‘The *Journal of Medical Microbiology* has also removed the Impact Factor from our home page. We support the Society’s decision to fight against the artificiality of the Impact Factor and reject its use that does more disservice than service to the scientists in this “publish or perish” era.’

Mark Harris and Paul Duprex. ‘The *Journal of General Virology* believes that Impact Factors are not a reliable or accurate indication of the quality of the science published in a journal. Now that accurate metrics on individual articles can be easily accessed, we believe that these provide a better way to assess both quality and impact. We therefore wholeheartedly support the Society’s decision to sign the DORA agreement.’

Martha E. Trujillo. ‘The *International Journal of Systematic and Evolutionary Microbiology* is the leading journal for the publication of novel microbial taxa. We applaud the decision of the Society to sign the DORA agreement and have also decided to remove the Impact Factor from our home page. We believe it does not reflect the quality of the work published.’

Nick Thomson and Stephen Bentley. ‘We purposely set up *Microbial Genomics* to record altmetrics for all our articles. There has been and almost certainly will continue to be intense debate around Impact Factor and its impact on the future direction of our field and those within it. We need to find new ways that are both practical and informative and so we welcome and support this initiative by the Society to sign up to DORA.’

